# Vitamin D concentration in the blood of women with twin pregnancies and in the umbilical cord blood of newborns in relation to environmental factors

**DOI:** 10.3389/fnut.2024.1433203

**Published:** 2024-09-18

**Authors:** Regina Ewa Wierzejska, Iwona Szymusik, Dorota Bomba-Opoń, Iga Rzucidło-Szymańska, Barbara Wojda, Robert Biskupski-Brawura-Samaha

**Affiliations:** ^1^Department of Nutrition and Nutritional Value of Food, National Institute of Public Health NIH – National Research Institute, Warsaw, Poland; ^2^Department of Obstetrics, Perinatology and Neonatology, Centre of Postgraduate Medical Education, Warsaw, Poland; ^3^Department of Obstetrics and Perinatology, National Medical Institute of the Ministry of the Interior and Administration, Warsaw, Poland; ^4^Department of Gynecology and Obstetrics, Institute of Medicine Collegium Medicum, Jan Kochanowski University, Kielce, Poland

**Keywords:** vitamin D, twin pregnancy, umbilical cord blood, newborns, vitamin D supplementation

## Abstract

**Background:**

There is a huge gap in the knowledge of the body’s nutrient resources in women with multiple gestations. Due to the increased demand hypothesis and taking into account common vitamin D deficits in women with singleton pregnancies, this issue should also be investigated in twin pregnancies. This study evaluated blood vitamin D concentration in women with twin pregnancies and in the umbilical cord blood of their newborns as well as analyzed environmental factors that may affect the level of this nutrient.

**Methods:**

The study included 56 women with twin pregnancies. Venous blood samples were collected from the women before delivery and umbilical cord blood at delivery to determine the total 25(OH)D concentration. The women were interviewed by a dietitian to collect data on their diet and lifestyle.

**Results:**

The average maternal 25(OH)D concentrations were 38.4 ± 11.0 ng/mL vs. 23.7 ± 6.1 ng/mL determined in the umbilical cord blood of the newborns. The concentration of 25(OH)D in the umbilical cord blood was strongly correlated with the concentration in the mother (*p* < 0.001). Vitamin D deficiency was found in 7% of women and 21% of newborns. Factors increasing the risk of too low 25(OH)D concentration in the mothers were age below 27 years (*p* = 0.002) and short duration of pregnancy (*p* = 0.011). In newborns, the risk factors included low maternal concentrations (*p* < 0.001) and delivery before 36 weeks of gestation (*p* = 0.008). The mean cord blood 25(OH)D levels were almost identical in both twins and amounted to 24.0 ± 6.1 ng/mL in the first-born and 23.4 ± 6.1 ng/mL in the second-born infant. Vitamin D supplementation was declared by 98% of the women, with 85% taking ≤2,000 IU vitamin D daily.

**Conclusion:**

Only a small percentage of women with twin pregnancies presented with vitamin D deficiency, which was probably related to the widespread supplementation of this nutrient. It can therefore be assumed that a dose of 2,000 IU vitamin D currently recommended for pregnant women may also be appropriate for twin gestations, although further research is required to validate this finding.

## 1 Introduction

With the observed increase in the number of multiple pregnancies, there is a need to verify to what extent the dietary recommendations for pregnant women are also appropriate for twin pregnancies. The amount of scientific data is currently insufficient to allow formulating any valid conclusions, but it can be assumed that the nutritional needs in twin pregnancy are higher due to the faster depletion of maternal reserves (1–4).

The body’s nutritional requirements for vitamin D are of particular interest because of their common deficiencies in singleton pregnancies (5–7). These deficits may theoretically be more pronounced in women pregnant with twins, but the data in this regard are still quite limited. A Spanish study showed that multiple pregnancy increases the risk of low vitamin D levels by more than six times (8). Taking into account the complete dependence of the fetus on maternal resources, this issue requires scientific analysis, although the impact of vitamin D status on the outcome of pregnancy and the condition of the newborn is still a matter of debate. Observational studies in women with singleton pregnancies indicate that low vitamin D levels increase the risk of preeclampsia, gestational diabetes, premature birth, small for gestational age (SGA) and worse anthropometric parameters of the newborn (9–12), but these relationships have not been clearly demonstrated in cyclical meta-analyses or reviews of randomized controlled trials (RCTs). In light of one such review, vitamin D supplementation by the mother reduced the risk of SGA and increased birth weight (13). In another review, due to the moderate quality of the evidence, the authors only highlight a potentially beneficial effect of vitamin D supplementation on reducing the risk of preeclampsia, gestational diabetes and low birth weight (14). The opposite conclusions have been drawn by the authors of the latest RCTs review, namely that vitamin D supplementation had no benefits in pregnancy and, in their opinion, low 25(OH)D level may only be a marker of adverse effects caused by other factors (12).

A very limited number of studies on the relationship between vitamin D concentration and gestational outcomes have been conducted in twin pregnancies. This gap needs to be addressed, as multiple pregnancy is associated with a higher risk of perinatal complications. In a study by Bodnar et al., the authors found that vitamin D concentrations above 30 ng/mL reduced the risk of preterm birth by 60%, compared to women with lower concentrations (15), while an Italian study showed that women with twin pregnancies with vitamin D levels below 30 ng/mL in the first trimester were more likely to develop hypertensive disorders of pregnancy compared to women with normal levels (16). In turn, Nakayama et al. (17) and Okah et al. (18) observed significantly higher levels of bone resorption markers in women with twin pregnancies compared to singleton pregnancies.

Most global guidelines on vitamin D consumption during pregnancy make no distinction between the requirements of women with singleton and multiple pregnancies. According to the European Food Safety Authority, the adequate intake for pregnant women is 15 μg vitamin D per day (19), while the American Institute of Medicine recommends a lower amount for all pregnant women—10 μg/day (20). Only Canadian experts from Alberta Health Services recommend separate dietary guidelines for women pregnant with twins and believe that the total intake of vitamin D from food and supplementation should amount to 30 μg (1,200 IU) per day (21). It is well known that achieving the recommended intake levels from foods alone is virtually impossible. Even in Finland, where fish consumption is much higher than in other countries, pregnant women consume just over 7 μg of vitamin D per day (22). Therefore, meeting the recommended intake levels requires supplementation, but these recommendations vary significantly from country to country. In the light of the consensus of global scientific organizations, in order to prevent rickets in children, pregnant women are recommended to supplement 600 IU (15 μg) vitamin D per day (23). On the other hand, in the publication “*WHO antenatal care recommendations for a positive pregnancy experience*” WHO concludes that vitamin D supplementation should only concern women with suspected deficiency and a dose of 200 IU (5 μg) per day is sufficient in such case (24). Contrary to that, the latest guidelines prepared by a group of Polish experts recommend 2,000 IU (50 μg)/day to be used in pregnancy (25).

The global number of studies on vitamin D status in women with twin pregnancies and their babies is very limited. Therefore, the aim of this study was to evaluate the concentration of vitamin D (expressed as 25(OH)D) in the blood of women with twin pregnancies and in the umbilical cord blood of the newborns and to analyze environmental factors that may affect the status of this vitamin.

## 2 Materials and methods

### 2.1 Study design

The study involved 56 women in twin pregnancies admitted for childbirth at the 1st Department of Obstetrics and Gynecology, Medical University of Warsaw and at the Department of Obstetrics, Perinatology and Neonatology, Centre of Postgraduate Medical Education in Warsaw, in the years 2021–2023. The exclusion criteria were age below 18 years, delivery before 32 weeks of gestation, non-Polish nationality and chronic liver and kidney diseases likely to affect vitamin D metabolism. In addition, pregnancies complicated by twin-to-twin transfusion syndrome or with congenital anomalies of either of the twins were also excluded. After the patients gave their informed consents to participate in the study, venous blood was collected before delivery and umbilical cord blood samples at delivery to obtain “mother-newborn” blood sets. The study was approved by the Bioethics Committee of the National Institute of Public Health, National Institute of Hygiene—National Research Institute in Warsaw under No. 6/2021.

### 2.2 Laboratory analysis and data collection

The blood samples were tested for total 25(OH)D levels using immunological methods (LIAISON® 25 OH Vitamin D TOTAL Assay; DiaSorin). The lower detection threshold for vitamin D is 4.0 ng/mL. The intraassay and interassay CV were <8 and <10%, respectively. The following criteria of maternal serum 25(OH)D concentration were used: optimal ≥30–50 ng/mL, suboptimal >20–30 ng/mL, deficiency ≤20 ng/mL, high >50 ng/mL (25–27). As for umbilical cord blood, ≥20 ng/mL was the recommended level and lower values were treated as vitamin D deficiency (20, 28).

Vitamin D intake by the patients (from fish, eggs, margarine—mandatorily fortified with vitamin D in Poland, butter, milk and dairy products) and calcium intake (from milk and dairy products) was evaluated during an interview conducted by a dietitian based on the validated Food Frequency Questionnaire. The interviewer also asked the women to list any vitamin D supplements they were taking and for how long. This included single-component and multivitamin preparations. If the patient listed a specific trade-name product, the dose of vitamin D was determined based on the particulars appearing on the packaging. If the trade name was missing, the amount of vitamin D supplemented by the patient was estimated based on the authors’ assessment of the composition of vitamin/mineral supplements intended for pregnant women (29). Patients were also asked to provide information on the course of pregnancy, body weight before pregnancy, weight gain during pregnancy, smoking, sun exposure and sociodemographic data. Gestational weight gain was assessed based on the recommendations for women pregnant with twins issued by the American Institute of Medicine (30). Patients’ characteristics are presented in Table 1.

**Table 1:** Pregnant women characteristics.

Number of women, *n*	56
Including:	
Monochorionic pregnancies, *n* (%)	25 (45)
Dichorionic pregnancies, *n* (%)	31 (55)
Age (in years), mean ± SD	31.6 ± 4.8
Including:	
Age ≥ 27 years, *n* (%)	49 (87)
Education, *n* (%)	
Higher	39 (70)
Other	17 (30)
Place of residence, *n* (%)	
City/town	52 (93)
Rural/village	4 (7)
Gravidity, *n* (%)	
Primipara	30 (54)
Multipara	26 (46)
Gestational age (in weeks), median (min–max)	36 (33–38)
Delivery after 36 weeks of gestation, *n* (%)	50 (89)
Due date season, *n* (%)	
Spring–summer	27 (48)
Autumn–winter	29 (52)
Child’s gender	
Male, *n* (%)	51 (46)
Female, *n* (%)	61 (54)
Maternal Body Mass Index (BMI) prior to conception, median (min–max)	22.6 (16.5–38.9)
Gestational weight gain, *n* (%)	
Low	28 (50)
Normal	22 (39)
Excessive	6 (11)
Gestational diabetes, *n* (%)	10 (18)
Hypertension, *n* (%)	4 (7)
Anemia, *n* (%)	19 (34)
Smoking during pregnancy, *n* (%)	2 (4)
Vitamin D supplementation, *n* (%)	55 (98)
Daily vitamin D intake	
With food (μg), median (min–max)	2,4 (0.4–9.1)
With food and dietary supplements (μg), median (min–max)	52.2 (1.2–154.6)
Calcium intake from milk and dairy products (mg), median (min–max)	632 (0.0–2,900)
Fish consumption (at least once a week), *n* (%)	22 (39%)
Avoiding sun exposure (women with due date in the summer season), *n* (%)	17 (63%)

### 2.3 Statistical analysis

This paper analyses the determinants of 25(OH)D levels in maternal blood and umbilical cord blood of newborns related to maternal characteristics in terms of socio-demographic characteristics, course of pregnancy, and lifestyle elements (including nutrition and vitamin D supplementation).

The following descriptive statistics were determined for the variables: percentage terms for qualitative variables, mean value ± standard deviation (SD) or median and range of variability for quantitative variables, depending on the normal distribution. Compliance with normal distribution was verified using the Kolmogorov–Smirnov test. The statistical significance of differences in average values of vitamin D concentration in two unpaired groups for data meeting the test assumptions was compared using the Student’s t-test, and in the remaining cases using the parametric Mann–Whitney test. In case of paired variables (comparisons between twins), the paired t-Student test was used. The frequency of analyzed events in the subgroups was compared using the chi-square test and Fisher’s exact test in case of small sample size. The prevalence of vitamin D deficiency in the newborns assigned to four groups on the basis of maternal vitamin D levels was compared using the chi-square test for trend.

The linear regression method was used to assess the relationship between the concentration of 25(OH)D in maternal blood and the umbilical cord blood of the newborns (regression coefficients are given with standard error); the coefficient of determination, showing the proportion of variance in the dependent variable (vitamin D concentration in newborn’s blood) which can be explained by the independent variable (maternal vitamin D level) was calculated. The value of the Pearson correlation coefficient is also presented.

Both parts of the analysis (of the mothers and the newborns) identified factors associated with vitamin D concentration (using multivariate linear models) and with having a vitamin D level lower than the recommended one (logistic regression). In these latter analyses, the dependent variable was defined as 25(OH)D concentration below 30 ng/mL for mothers and below 20 ng/mL for newborns. The explanatory variables included maternal characteristics and pregnancy data. The analysis was conducted in two steps. In the first step (by simple logistic regression), the appropriate odds ratios (OR) and their 95% confidence intervals (95% CI) were calculated, determining the strength and statistical significance of association of individual factors with the dependent variable. To control for correlations between explanatory variables, the second step of the analysis was conducted using a multivariate logistic regression model. In this step, the explanatory variables were those factors that showed a statistically significant relationship with the dependent variable in the first step of the analysis. To assess the quality of the resulting model, the Receiver Operating Characteristic (ROC) curve was plotted and the Area Under Curve (AUC) index and its 95% confidence interval were calculated.

The significance level of 0.05 was adopted for all statistical analyses. They were carried out using SPSS software version 12.0 PL.

## 3 Results

### 3.1 Vitamin D concentration in maternal blood

**Figure 1:**
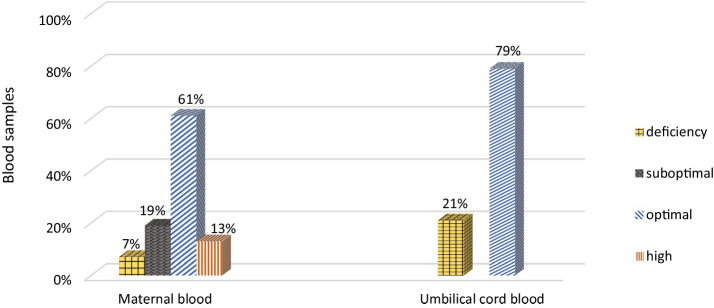
Vitamin D status in maternal and umbilical cord blood.

The mean concentration of 25(OH)D in maternal blood was quite high and amounted to 38.4 ± 11.0 ng/mL (range 15.1–64.4 ng/mL). There was no statistically significant difference between 25(OH)D concentrations in women with monochorionic and dichorionic pregnancies (mean 37.9 ± 10.0 vs. 38.7 ± 11.7 ng/mL). Vitamin D deficiency (≤20 ng/mL) was found in only 7% of the mothers (Figure 1).

A multivariate linear regression model indicated a statistically significant relationship between the place of residence, mother’s age and duration of pregnancy with the 25(OH)D concentrations. Women from urban areas had 10.4 (standard error, SE = 4.3) ng/mL higher levels of 25(OH)D (*p* < 0.015) than women living in the countryside. Women aged ≥27 years had higher 25(OH)D levels than younger women by 15.9 (SE = 3.6) ng/mL (*p* < 0.001), and women delivering after completed 36 weeks of gestation had higher 25(OH)D levels by 12.5 (SE = 3.6) ng/mL (*p* < 0.001) than women delivering before 36 weeks of gestation.

Factors associated with 25(OH)D concentration below 30 ng/mL included the duration of pregnancy, delivery before 36 weeks of gestation, education, age and height of the mother (Table 2).

**Table 2:** Factors statistically significantly associated with 25(OH)D levels below 30 ng/mL.

Explanatory variable	OR	95% CI	Statistical significance (*p*)
Duration of pregnancy (in weeks)	0.384	0.173–0.855	0.019
Delivery ≥36 weeks	0.050	0.005–0.477	0.009
Higher education	0.246	0.070–0.864	0.029
Maternal height ≥ 163 cm	0.883	0.781–0.998	0.046
Maternal age ≥ 27 years	0.038	0.004–0.351	0.004

In the second step of the analysis (multi-factor model) the only two remaining factors were duration of pregnancy and maternal age. The risk of 25(OH)D levels falling below 30 ng/mL was more than three times lower (OR = 0.290, 95%CI: 0.112; 0.751, *p* = 0.011) with each additional week of pregnancy (weeks 33 through 38), and maternal age ≥ 27 years reduced the risk 48 times (OR = 0.021, 95%CI: 0.002; 0.242, *p* = 0.002).

### 3.2 Vitamin D concentration in neonate cord blood

The mean concentration of 25(OH)D in the umbilical cord blood of the newborns was 23.7 ± 6.1 ng/mL (range 10.6–39.5 ng/mL). Similar to maternal blood findings, no statistically significant differences were observed for 25(OH)D concentrations in the cord blood from monochorionic and dichorionic pregnancies (mean 24.0 ± 5.3 vs. 23.5 ± 6.7 ng/mL). The mean concentration of 25(OH)D in the umbilical cord blood was almost identical for both twins and amounted to 24.0 ± 6.1 ng/mL in the first born and 23.4 ± 6.1 ng/mL in the second born baby, without differentiating between the type of pregnancy (monochorionic vs. dichorionic). There were also no statistically significant differences in 25(OH)D levels between newborns with higher and lower birth weights (mean 23.5 ± 6.0 vs. 23.9 ± 6.2 ng/mL). Finally, no differences with respect to 25(OH)D concentration were found depending on the child’s gender (median 23.5 ng/mL in girls and 23.2 ng/mL in boys).

**Figure 2:**
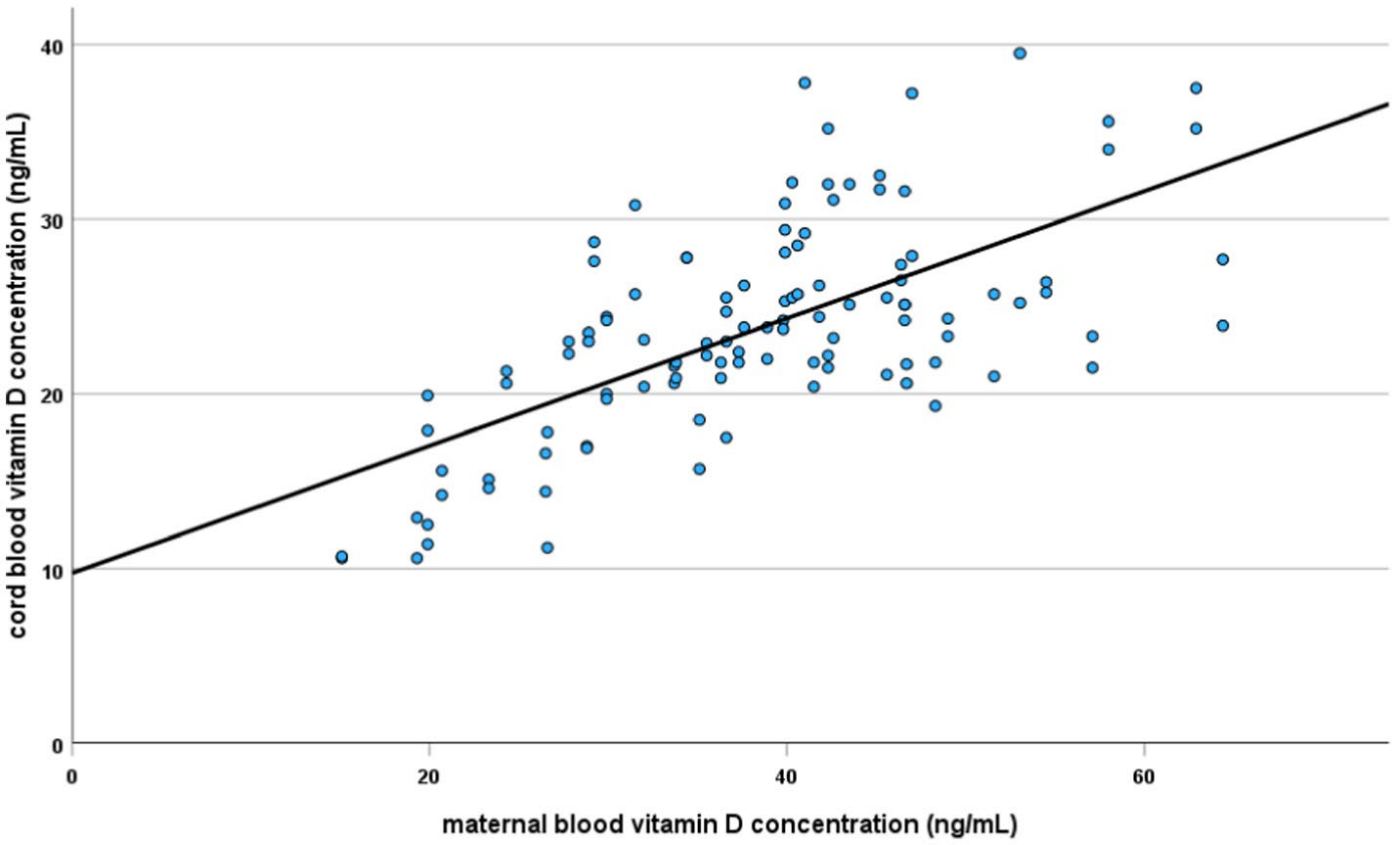
Vitamin D concentration in maternal blood vs. umbilical cord blood.

Vitamin D deficiency (<20 ng/mL) was present in 21% of the newborns (Figure 1). In each analyzed case (mother vs. newborn) the concentration of 25(OH)D in the umbilical cord blood was lower than in the mother. A strong, statistically significant relationship was demonstrated between the concentration of vitamin D in maternal and umbilical cord blood. An increase by 1 ng/mL in maternal blood corresponded to a 0.34 (SE = 0.04) ng/mL increase in cord blood levels (*p* < 0.001). Maternal vitamin D levels explain approximately 43% of the variability in newborn concentrations (Pearson correlation coefficient = 0.653) (Figure 2).

The percentage of the newborns with vitamin D deficiency was observed to decrease with the increase in maternal levels. All neonates born to vitamin D deficient mothers had vitamin D deficiency. Half of the newborns of mothers with suboptimal concentrations had such deficits. Only 5% of the newborns of mothers with optimal 25(OH)D concentrations had vitamin D deficiency, while none of the newborns of mothers with high 25(OH)D levels (>50 ng/mL) had deficient levels. This trend was statistically significant (*p* < 0.001).

The linear multivariate model shows that, in addition to maternal levels, there was a statistically significant relationship between umbilical cord blood levels of 25(OH)D and the season of the year, maternal BMI and calcium intake. The concentration of 25(OH)D in newborns born between May and September, whose mothers did not avoid sun exposure, was higher by 2.9 (SE = 0.9) ng/mL than in the newborns born in other months of the year (*p* < 0.001). The concentration of 25(OH)D in newborns of mothers with high pre-gestational body weight (BMI ≥ 25) was 2.4 (SE = 0.9) ng/mL lower than in newborns of mothers with normal body weight (*p* = 0.008) and 5.4 (SE = 1.5) ng/mL lower than in newborns of underweight mothers (BMI <18.5) (*p* < 0.001). An increase in maternal calcium intake by 100 mg per day corresponded to an increase in cord blood 25(OH)D concentration by 0.2 (SE = 0.1) ng/mL (*p* = 0.006).

As for the risk factors for vitamin D deficiency in newborns (<20 ng/mL), they are presented in Table 3.

**Table 3:** Factors statistically significantly associated with 25(OH)D levels below 20 ng/mL.

Explanatory variable	OR	95% CI	Statistical significance (p)
Maternal blood 25(OH)D concentration	0.772	0.695–0.858	<0.001
Delivery ≥ 36 weeks	0.033	0.006–0.164	<0.001
Maternal age ≥ 27 years	0.146	0.045–0.479	0.001
Higher education of mother	0.333	0.131–0.849	0.021
Maternal height (in cm)	0.875	0.797–0.960	0.005
Maternal BMI prior to conception	1.128	1.023–1.244	0.016
Daily maternal vitamin D intake with food and dietary supplements (μg)	0.975	0.955–0.996	0.020
Calcium intake from milk and dairy products (100 mg)	0.905	0.919–1.000	0.045

**Figure 3:**
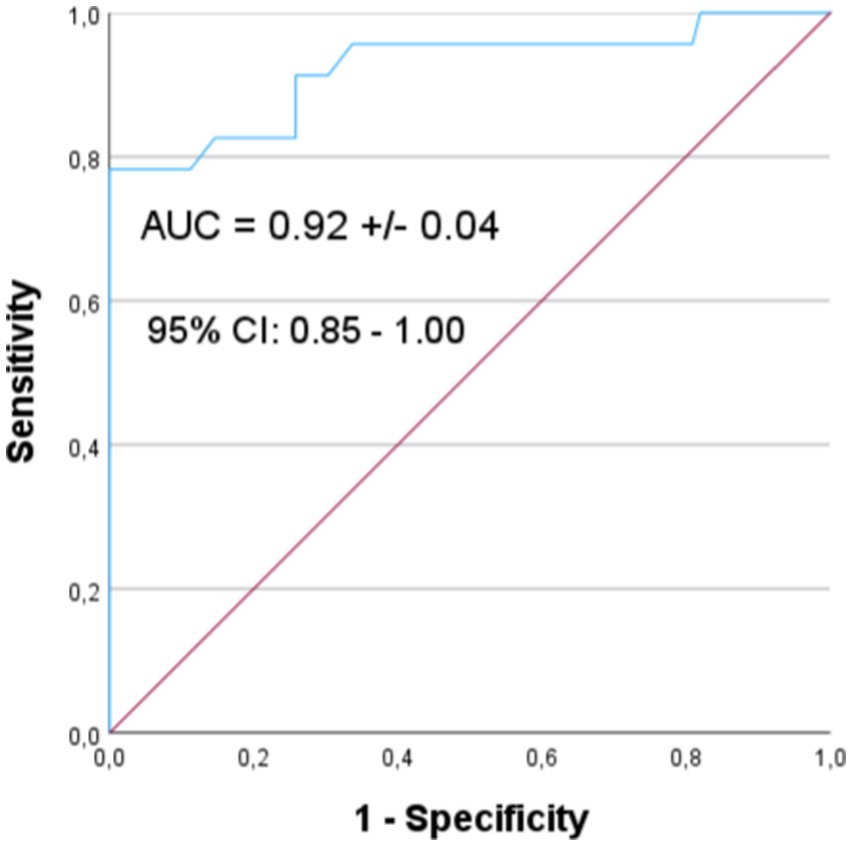
ROC curve for the multivariate model of vitamin D deficiency in newborns.

A multivariate logistic regression analysis showed that of all the above-mentioned risk factors for vitamin D deficiency in newborns, only maternal levels and the duration of pregnancy were statistically significant. The risk decreased by 21% with an increase in maternal concentration by 1 ng/mL, (OR = 0.79, 95%CI: 0.71; 0.88, *p* < 0.001). The risk was over 14 times lower in babies born after 36 weeks of gestation compared to shorter duration of pregnancy (OR = 0.07, 95%CI: 0.01; 0.50, *p* = 0.008). The resulting model has very good prognostic value, allowing to correctly classify 22 out of 23 newborns with vitamin D deficiency based on only two factors (with 95.7% sensitivity and 66.3% specificity). The ROC curve is shown in Figure 3. The AUC is 0.92 ± 0.04 [95%CI: 0.85–1.00].

### 3.3 Vitamin D concentration vs. maternal vitamin D intake/supplementation

The intake of vitamin D with food was low—median 2.4 μg per day (min 0.4 μg – max 9.1 μg). Almost all (98%) of the study population supplemented vitamin D (single-component vitamin D preparations or vitamin/mineral supplements) with doses ranging from 200 to 6,000 IU (5–150 μg). Within this group, 22% of the women took less than 2,000 IU (<50 μg) of vitamin D daily, 63% of the women took 2,000 IU, 11% of patients declared taking >2,000–4,000 IU (>50–100 μg), and the remaining 4% used higher doses. Only one patient (2%) started supplementation only in the third trimester of pregnancy, and two patients (4%) in the second trimester. The remaining patients used vitamin D supplements regularly since the beginning of pregnancy. There was no statistically significant relationship between the supplemented dose of vitamin D and the level of this nutrient in the mother. Maternal concentration range in women taking 2,000 IU vitamin D was very wide (19.3–64.4 ng/mL), with 74% of them showing optimal levels (≥30 ng/mL). Although 25(OH)D concentrations below 30 ng/mL were more common in women supplementing less than 2,000 IU a day compared with those taking higher doses (31% vs. 26%) and such women had lower mean 25(OH)D concentrations (34.0 ± 9.3 ng/mL vs. 39.7 ± 11.1 ng/mL), these differences were not statistically significant.

As far as newborns are concerned, babies born to mothers taking <2,000 IU vitamin D were statistically significantly more likely to have a deficiency compared to babies born to mothers taking higher doses (35% vs. 16%) (*p* = 0.046). The differences in 25(OH)D cord blood levels measured in mothers supplementing <2,000 IU vs. ≥2,000 IU were not statistically significant (median 21.8 ng/mL vs. 23.9 ng/mL, respectively).

## 4 Discussion

Assuming that the currently recommended thresholds should be equally applied to women with singleton and twin pregnancies, approximately 25% of women participating in our study presented with suboptimal 25(OH)D levels (<30 ng/mL). This is slightly above the figure reported by Zgliczyńska et al. in the only other study in Polish women in twin gestations published to date (11.5%) (31). Thirteen percent of the mothers had concentrations exceeding 50 ng/mL, which is considered high but still within the safety limits, as the risk of vitamin D toxicity is believed to occur at concentrations above 100 ng/mL (5, 25). Equally limited data is available from other countries, and the comparison of the results is still more difficult due to differences in the criteria used to assess vitamin D status. In the USA, 40% of women in twin pregnancies had concentrations below 30 ng/mL based on the same reference threshold as we used in our study (15). Similar findings were also reported in China (40%) (32). In an Austrian study in a cohort of singleton and twin pregnancies, as many as 94% of women had concentrations below 30 ng/mL on the day of delivery (26). Therefore, the relatively low percentage of vitamin D deficient women in our study, in comparison to other countries, was probably related to the widespread vitamin D supplementation as declared by almost all women participants (98%). Similar supplementation rates (96%) were reported among women in twin gestations in the above cited Polish study (31). Also, almost 80% of the women in our study used at least 2,000 IU vitamin D a day, while in the Austrian study only 66% supplemented vitamin D and at a much lower doses (median 400 IU per day) (26). Many studies confirm that supplementation with 400 IU vitamin D by pregnant women is not sufficient to achieve the recommended blood levels (26, 33, 34). Higher amounts of vitamin D supplemented by Polish women is likely related to the increase in the content of this nutrient in vitamin and mineral supplements for pregnant women which has been observed in the recent years. In 2014, only 5% of dietary supplements available on the Polish market contained 2,000 IU vitamin D compared to 50% already in 2019 (29, 35).

We did not find a statistically significant relationship between the concentration of 25(OH)D in maternal blood and its intake with food or supplements. In case of consumption with food, this is an expected finding, since none of the participants supplied sufficient amounts of vitamin D through their diet to affect the blood levels, which is consistent with the results of other studies, including those involving Polish population (7, 26). On the other hand, the lack of correlation between vitamin D concentration and its supplementation could be explained by the fact that only one of the participants did not take vitamin D supplements. Still, at a daily dose of 2,000 IU, the concentration range was very wide, and this may result from many factors. Firstly, the body’s response to supplementation varies between individuals (34, 36–38). Secondly, we have no knowledge on the levels of vitamin D prior to conception. Mothers who had higher levels of vitamin D in the preconception period (e.g., due to previous supplementation) could achieve still higher concentrations via continuous supplementation during pregnancy than those who conceived already with a deficiency. We are also not sure (despite patient declarations) whether each of the women used vitamin D supplements on a regular basis. At the same time, some Polish experts suggest that pregnant women should have their 25(OH)D concentrations determined before starting supplementation in order to adjust the optimal dose (39), and others claim that supplementation should always be supported by the results of blood tests (25, 40). In the case of the women participating in our study 25(OH)D concentrations were measured only before delivery.

In our study, the 27 years age threshold proved to be a factor that had significant correlation with vitamin D concentrations, with more favorable findings in mothers who were at least 27 years old. Justifying this correlation is not easy as the median dose of the supplemented vitamin D did not differ in both age groups. Still, in the younger group, 43% of women supplemented less than 2,000 IU vitamin D a day and none of them used a higher dose, whereas only 20% of the women in the ≥27 age group supplemented less than 2,000 IU and 18% exceeded this dose. One of the possible explanations is that it might have taken more time for the women in the older age group to conceive and some of them may have sought infertility treatment and therefore regularly took vitamin D in the preconception period. However, no such data was collected from the participants. It is also possible that older maternal age may be associated with better general health awareness, translating into healthier lifestyle and, theoretically, higher body pool of vitamin D. Due to the small study sample, however, this finding should be approached with caution, especially since the opposite was observed in one of the previous studies, where older mothers had a higher risk of vitamin D deficiency (41).

With regard to the umbilical cord blood levels, studies clearly show a correlation between the maternal and neonate levels both in singleton (7, 42–44) and twin (32, 45–47) pregnancies. Such correlation was also found in our study. The mean concentration of 25(OH)D in umbilical cord blood was lower than the mean concentration in the mothers, amounting to 62% of maternal levels. This is consistent with many studies, where the concentration in the newborn reached 48–89% of that measured in the mother (32, 41, 47). However, studies conducted in Australia and India on women with twin pregnancies demonstrated the opposite relationship, i.e., higher umbilical cord blood levels (45, 46). Due to lower vitamin D concentrations in umbilical cord blood, vitamin D deficiency in our study occurred three times more often in the newborns than in the mothers, but overall it affected only about 20% of the newborns. This percentage varied significantly depending on the maternal levels. The percentage of vitamin D deficient newborns decreased with increasing maternal levels, which is consistent with the conclusions from other studies to date (5, 28, 44). Our study also confirmed the hypothesis on the absence of differences in the concentrations of vitamin D in the umbilical cord blood of the twin neonates, which is also supported by the findings reported by Novakovic et al. and Goswami et al. (45, 46).

Lower 25(OH)D concentrations found in the neonates born in the autumn and winter season also confirm the previous observations (7, 32, 48). As for the relationship between vitamin D levels and the BMI status of the mothers, previous studies have shown significantly lower concentrations of vitamin D in pregnant women with excessive body mass index (41, 49, 50). In our study, the relationship between vitamin D concentration and maternal BMI was demonstrated for umbilical cord blood levels. Whereas the lower risk of vitamin D deficiency for the mother and newborn in case of longer duration of pregnancy could indicate that the levels of vitamin D increase with the duration of pregnancy, which has already been shown in some studies, regardless of maternal supplementation (51, 52). We did not analyze the possible impact of low vitamin D levels on the duration of pregnancy, as the study concerned twin pregnancies, in which the decision to deliver is often made due to medical reasons. In case of uncomplicated twin pregnancies, the current guidelines recommend to deliver dichorionic twins between 37 and 38 weeks, while monochorionic twins between 36 and 37 weeks of gestation. This recommendation is based on the findings from multicentre studies, where the lowest rates of neonatal complications were recorded in the weeks cited, although for all monochorionic pregnancies such delivery is still premature by definition (53).

It is also worth having a brief discussion about how our results compare to other Polish studies in women with singleton pregnancies. In our study, the percentage of twin pregnancies with optimal vitamin D concentrations (74%) was higher than in studies among women in singleton pregnancies (11–30%) (7, 54–56), while the percentage of vitamin D-deficient women (7%) was lower (31–50%) (7, 55). However, it should be noted that the cited studies were conducted in the years 2013–2017, when the issue of vitamin D supplementation was not addressed by the media and the medical community as much as it is today. In the latest Polish study (2023), only 7.5% of women in singleton pregnancies had vitamin D levels below 30 ng/mL (31).

As far as we know, this is the first study in Poland and only the fifth in the world (32, 45–47) to include women with twin pregnancies and their newborns. Therefore, it is a vital contribution to the still limited knowledge on vitamin D status in mothers and twin babies. The results of our study as well as the other Polish study on twin pregnancies referred to above (31) indicate that by taking 2,000 IU of vitamin D daily, most women are able to achieve the recommended blood levels.

Nevertheless, there are certain limitations which should be taken into account when interpreting the results. First of all, the measurements of maternal 25(OH)D levels were solely performed on the day of delivery, which does not mean that these levels were maintained throughout all or most of the pregnancy. Therefore, we do not know whether the newborns had a relatively constant supply of vitamin D in their fetal life. Another important issue that has recently been raised involves different methods of measuring vitamin D which yield different results. In one study in which measurements were made using two methods, one of which was LIAISON® immunoassay (which was also used in our study) and the other was liquid chromatography–tandem mass spectrometry (LC–MS/MS) recognized as the gold standard, the authors reported significant differences in the measured vitamin D levels. In women with twin pregnancies, the median 25(OH)D concentration determined by the immunoassay method was almost 14 ng/mL (approximately 30%) lower than the median concentration measured using the LC–MS/MS method (31). Therefore, if we were to assume that our measurements were underestimated, then we could also assume that none of the women and newborns had vitamin D deficiency. On the other hand, in a study among women with singleton pregnancies, where both of the above-mentioned methods were also used, the concentration in cord blood determined by the immunoassay method was 13.5 ng/mL (approximately 57%) higher than the concentration measured by LC–MS/MS. According to the authors, the immunoassay method overestimates the concentration of vitamin D in umbilical cord blood, but not in maternal blood (57). Other experts have also discussed to possible overestimation of vitamin D levels determined by immunoassays as these measurements detect 25(OH)D together with vitamin D epimers which are probably biologically inactive (5, 41, 58, 59).

Another limitation of our study is the relatively small sample size. One of the reasons is that women in twin pregnancies constitute a disproportionately smaller population than singleton pregnancies. It is also worth emphasizing that the study was carried out in two separate tertiary perinatology centers in Warsaw which also run separate outpatient clinics for multiple pregnancies. It can therefore be assumed that women had access to better antenatal education, including diet and vitamin supplementation. Thus, the study group may not adequately reflect vitamin D status of patients from smaller centers, both in terms of concentration and supplementation.

## 5 Conclusion

Our study showed that most women in twin pregnancies had optimal vitamin D levels at delivery and only 7% had vitamin D deficiency. This favorable status was probably related to the widespread supplementation of vitamin D. Since most of the patients supplemented 2,000 IU of vitamin D daily, it can therefore be assumed that a dose of 2,000 IU of vitamin D currently recommended for pregnant women is also appropriate for twin gestations, although further research is required to validate this finding.

## Data Availability

The raw data supporting the conclusions of this article will be made available by the authors, without undue reservation.
